# What is the current status of childhood obesity in Saudi Arabia?: Evidence from 20,000 cases in the Eastern Province: A cross-sectional study

**DOI:** 10.1097/MD.0000000000029800

**Published:** 2022-07-08

**Authors:** Waleed Albaker, Rim Saklawi, Sulaiman Bah, Kamaluddin Motawei, Basem Futa, Mohammed Al-Hariri

**Affiliations:** a Department of Medicine, College of Medicine, Imam Abdulrahman Bin Faisal University, Dammam, Saudi Arabia; b Lebanese American University, Lebanon; c Department of Public Health, College of Public Health, Imam Abdulrahman Bin Faisal University, Dammam, Saudi Arabia; d Department of Anatomy, College of Medicine, Imam Abdulrahman Bin Faisal University, Dammam, Saudi Arabia; e Johns Hopkins Aramco Healthcare, Saudi Arabia; f Department of Physiology, College of Medicine, Imam Abdulrahman Bin Faisal University, Dammam, Saudi Arabia.

**Keywords:** blood pressure, children, obesity, Saudi Arabia

## Abstract

The prevalence rate of those overweight, as well as obesity among children in Saudi Arabia, keeps rising. The aim of the study was to estimate childhood obesity in the Eastern Province, Saudi Arabia.

Over the period 2016 to 2017, a cross-sectional campaign was carried out in the Eastern Province of Saudi Arabia. Data were collected from over 20,000 boys’ and girls’ schools. The campaign collected data on birthday/age, weight, height, sex, district in which the school is located, level of education, and blood pressure level.

The findings from the present study indicated a prevalence of 25.7% for overweight and obesity among high school-age students. More importantly, ≈35% of the study’s students have either elevated blood pressure or hypertension. The significant predictors of childhood obesity were education level, age, glucose level, and blood pressure.

The children in higher school levels originally from the Eastern Province had a high prevalence of overweight and obesity. Recommendations are made on the need of regular screening program among school-age children, as well as to continue raising awareness about childhood obesity.

## 1. Introduction

Childhood obesity is one of the most common health problems in the world due to its rising trend.^[1]^ According to the World Health Organization, >1.9 billion people were overweight in both developing and developed countries in 2016 with >340 million children and adolescents (5–19 years) being overweight or obese.^[2]^

The global number of overweight or obese newborns and young children is expected to reach 70 million by 2025.^[3]^ Overweight and obesity have been found to be significantly correlated with an increased risk of several health disorders, such as cardiovascular disease, psychological disturbance, metabolic syndrome, type 2 diabetes, fatty liver disease, and premature death.^[4]^ Childhood obesity is of particular concern as it has increased globally, and it could have adverse health impact in adulthood.^[5]^

Over the past 3 decades, several studies have been conducted on childhood obesity in different regions in Saudi Arabia. In a study carried out by El Mouzan et al (2010), the aim was to estimate the national prevalence of obesity and of being overweight in children in Saudi. This study found that Saudi Arabia’s level was intermediate, between those of developing and developed countries.^[6]^ In another study carried out in 2015 to 2016 based on data from 3613 school-aged children from different regions of Saudi Arabia, the estimated prevalence of obesity and of being overweight was 21.5%.^[7]^ The same study stated that obesity began to rise from age 10 and continued to increase and develop in significance and complexity until the age of 19 years.^[7]^

Overall, Saudi Arabia currently ranks among the countries with the highest prevalence of childhood obesity.^[8]^ Moreover, obesity-related health consequences among children in Saudi Arabia keep rising and, according to a national study, are significantly linked to cardiometabolic risk factors, which in turn impact the possibilities of being associated with other social and psychological outcomes.^[9]^

To achieve the Saudi Vision by 2030, the Ministry of Health aims to fulfill multiple strategic objectives that promote health status and support efforts toward an efficient disease-free society, including strategic objective number 13, which is targeted to lower obesity among Saudis.^[10]^ The first step to controlling the obesity among children is to accurately evaluate its prevalence and to tailor evidence-based effective intervention programs. Moreover, as far as we know, there is no recent report describing the existing childhood obesity prevalence in the Eastern Province, Saudi Arabia. According to the General Authority for Statistics, the Eastern Province has the highest average monthly income per household in Saudi Arabia.^[11]^ As various reports have documented that obesity is frequently associated with income growth, it is of interest to learn about the current rate of childhood obesity in the province.^[12,13]^ Thus, the aim of the present study was to provide a recent estimate of childhood body weight and determine the trend in childhood obesity in the Eastern Province, Saudi Arabia.

## 2. Methods and materials

From 2016 to 2017, a cross-sectional campaign was carried out in schools in the Eastern Province of Saudi Arabia. The campaign was carried out by the Saudi Diabetes and Endocrine Association in collaboration with the Ministry of Education, Eastern Province Branch.

Inclusion criteria were as follows: Saudi, with subjects aged 6 to 19 years of any sex. Participants with any acute or chronic health condition or amputated body parts were excluded from the study, since these conditions could affect their body mass index (BMI).

The campaign collected data on birthday/age, weight, height, sex, district in which the school is located, level of education, history of diabetes, pressure level, glucose level, frequency of exercise, and comorbidities. Blood pressure was measured (after 5 minutes of rest) using an automatic BP monitor using Dinamap.^[14]^ Weight and height were measured using a portable stadiometer (Seca 704; Seca, Hamburg, Germany). Participants were requested to “remove their shoes and wear light clothing before taking their measurements.” The height of the students was documented to the nearest 0.1 cm, and the weight was calculated to the nearest 0.1 kg.^[15]^

Two blood pressure records were taken “after 5 minutes of rest” using an automatic monitor (Omron M6 Comfort IT), and the mean of the blood pressure recordings was considered for the data analysis.^[14]^

Data were also collected on the names and telephone numbers of the students. The data on weight and height were used to calculate BMI. Children were classified based on BMI as underweight (BMI: <18.5), normal (BMI: 18.5–24.9), overweight (BMI: 25–29.9), or obese (BMI: ≥30).^[14]^ The data collected were cleaned and merged together into one file, giving each record a unique serial number. The data cleaning involved the following aspects:

Assessment of the accuracy of the BMI variable. After some were found to be inaccurate, the BMI variable was recalculated for all the cases. Cases with implausible values were eliminated. The BMI variable was grouped into standard obesity categories.
The blood pressure values were coded into standard hypertension categories.
Retention of the age variable as reported. The implausible age of 1 was dropped (2 cases).
Retention of the blood glucose level as reported. Even though some of the values were found to be very high (over 500 mg/dL), there was no valid reason to drop them.

The following variables were dropped from the analysis:

The birthday variable, as age could not be easily calculated from it for several reasons, including lack of uniformity in reporting the birthdays.
The frequency of exercise, as it was written in free text.
Comorbidities, as they were written in free text after the data cleaning, and the final file consisted of little over 20,000 cases.

Informed signed written parental consent and child assent were obtained from all participants. The study was approved by the Ethics Committee of the Imam Abdulrahman Bin Faisal University, Institutional Review Board (IRB-2021-01-296).

### 2.1. Statistical analysis

Data were analyzed using the MedCalc, PAST, and SSP softwares. Normality distribution was assessed using the Shapiro–Wilk test. The distribution of the continuous variables was presented using the median and interquartile range (IQR). Categorical variables were summarized using percentages. Associations between categorical variables were assessed using the χ^2^ test. Multiple regression was used to assess the predictors of BMI. For all the analyses, the significance level was set at 5% (α = 0.05).

## 3. Results

The frequency distribution of the categorical variables in the study population is shown in Table 1. Data were collected from over 20,000 boys’ and girls’ schools from different districts of the Eastern Province. The school population is made up of 57.5% boys. They are roughly equally distributed between primary (34.3%), intermediate (33.5%), and secondary (32.1%) levels. In total, the students covered in the campaign come from 14 districts. Of these districts, a little over half (50.5%) come from the 4 districts of Al-Thuqbah, Madinat Al Umal (central business district), Al Jisr, and Iskan (Khobar housing). These are all in the south and are of relatively lower income compared to higher income districts in the North of Khobar. The frequency distribution of the BMI classes shows a skewed distribution with 34.2% being underweight and 40% being of normal weight. About a quarter of the students (25.7%) are obese or overweight. For blood pressure, data are not stated for close to a third of the students. The frequency distribution of the blood pressure classes in Table 1 shows that 35.3% of the students are of normal blood pressure. Students with either elevated blood pressure or hypertension amount to 34.8%.

**Table 1:** Frequency distribution of categorical variables, school health campaign.

Variable	Categories	Number	Percentage
Sex	Male	11537	57.50
	Female	8536	42.50
	Total	20073	100.00
School level	Primary	6895	34.30
	Intermediate	6734	33.50
	Secondary	6444	32.10
	Total	20073	100.00
District	Al-Thuqbah	4019	20.00
	Madinat Al Umal	3129	15.60
	Al Jisr	2996	14.90
	Iskan	2206	11.00
	Al Aqrabiyah	1933	9.60
	Al Khobar Al Shamalia	1465	7.30
	Raka	748	3.70
	AIR-BASE	590	2.90
	Al Tahliyah	574	2.90
	Olaya	399	2.00
	Khobar Al Janubiyah	336	1.70
	Al Hizam Al Thahabi	283	1.40
	Tahliyah	158	0.80
	Al Hada	130	0.60
	Not stated	1107	5.50
	Total	20073	100.00
BMI class	Underweight (BMI < 18.5)	6862	34.20
	Normal (18.5 ≤ BMI ≤ 24.9)	8039	40.00
	Overweight (25.0 ≤ BMI ≤ 29.9)	2974	14.80
	Obese-1 (30.0 ≤ BMI ≤ 34.9)	1334	6.60
	Obese-2 (35.0 ≤ BMI ≤ 39.9)	529	2.60
	Obese-3 (BMI ≥ 40.0)	335	1.70
	Total	20073	100.00
Blood pressure class	Normal <120/<80 mm Hg	7083	35.30
	Elevated <120–129/<80 mm Hg	1995	9.90
	Hypertension stage I 130–139/80–89 mm Hg	3037	15.10
	Hypertension stage II ≥140/ ≥90 mm Hg	1962	9.80
	Not stated	5996	29.90
	Total	20073	100.00

Blood pressure classes.^[14]^

BMI = body mass index.^[15]^

The characteristics of the numeric variables in the study are shown in Table 2. For this young student population, the median age is 14 (IQR: 12–16) years, median weight is 49.6 (IQR: 36.5–63.5) kg, median height is 1.54 (IQR: 1.4–1.63) m, and the median BMI is 20.54 (IQR: 17.46–25.2) kg/m^2^.

**Table 2:** Characteristics of the numeric variables, school health campaign.

	Weight (kg)	Height (m)	BMI (kg/m^2^)	Age (y)
Median	49.6	1.54	20.54	14
Lower quartile, Q1	36.5	1.4	17.46	12
Upper quartile, Q3	63.5	1.63	25.2	16

BMI = body mass index.

**Figure 1.**
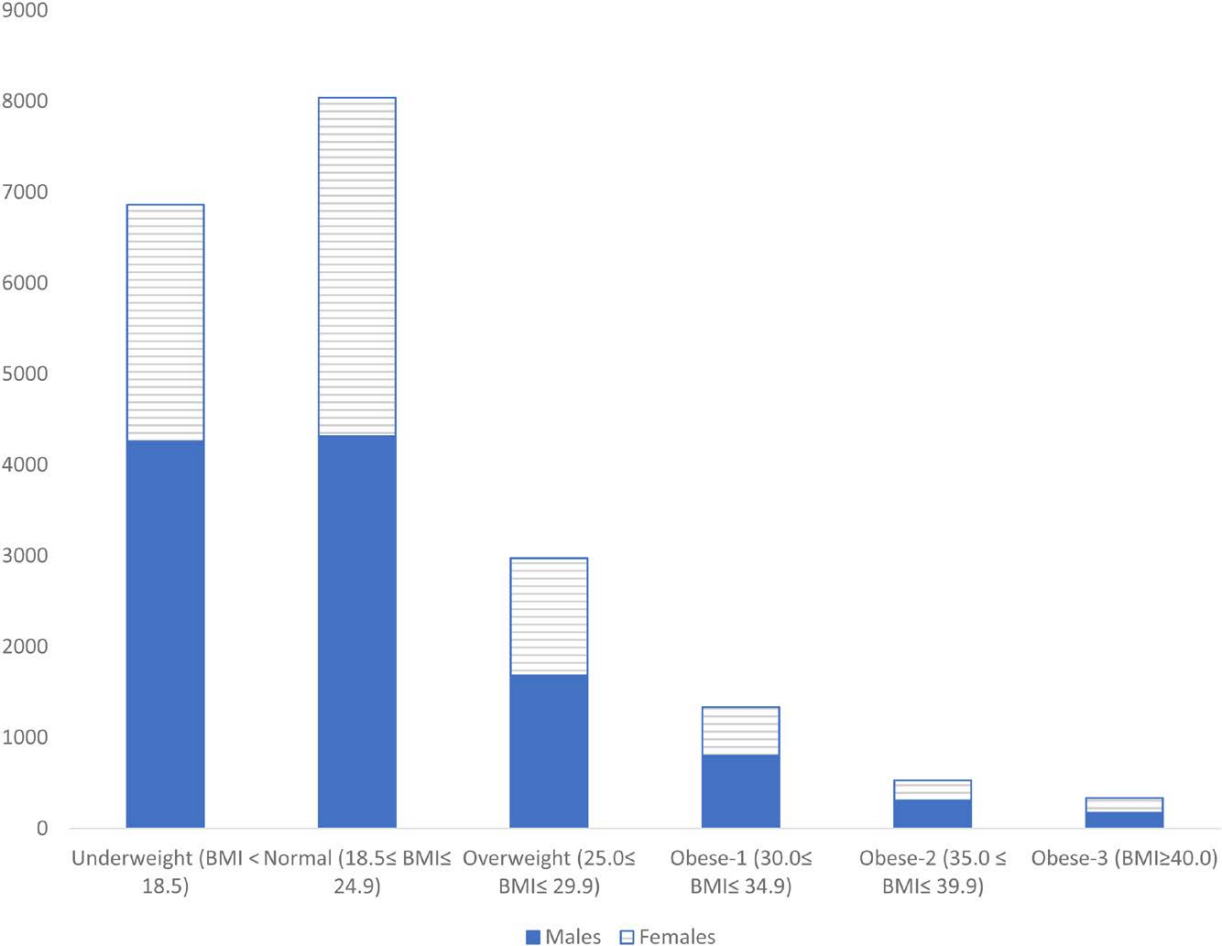
Comparison of the distribution of BMI classes among males and females, school health campaign. BMI = body mass index.

The association between sex and BMI classes is shown in Table 3. The table shows that for both males and females, the prevalence rates decrease with increase in the BMI level above the normal range (overweight and obese levels). The association is significant as *P* < .0001. This distribution of males and females within each BMI class is shown in Figure 1, and proportions are shown in Table 4. The table shows that for underweight, there is statistical difference between the proportions of males and females, with the proportion of males being higher. For normal weight, there is also statistical difference between the proportions of males and females but with the proportion of females being higher. For both overweight and obese categories, there is no statistical difference between the proportions of males and females.

**Table 3:** Association between sex and BMI classes, school health campaign.

BMI class	Male	Female	χ^2^(df)	*P* value
Underweight (BMI < 18.5)	4258	2604	114.657 (5)	<.0001
Normal (18.5 ≤ BMI ≤ 24.9)	4319	3720		
Overweight (25.0 ≤ BMI ≤ 29.9)	1682	1292		
Obese-1 (30.0 ≤ BMI ≤ 34.9)	800	534		
Obese-2 (35.0 ≤ BMI ≤ 39.9)	306	223		
Obese-3 (BMI ≥ 40.0)	172	163		
Total	11,537	8536		

BMI = body mass index, df = degree of freedom.

**Table 4:** Difference between male and female proportions according to BMI classes, school health campaign.

BMI classes	Male (n = 11,537)	Female (n = 8536)	z	*P* value
Underweight (BMI < 18.5)	36.9%	30.5%	9.453	<.001
Normal (18.5 ≤ BMI ≤ 24.9)	37.4%	43.6%	8.783	<.001
Overweight (25.0 ≤ BMI ≤ 29.9)	14.6%	15.1%	1.075	.283
Obese (BMI ≥ 30)	11.1%	10.8%	0.672	.502

BMI = body mass index.

**Figure 2.**
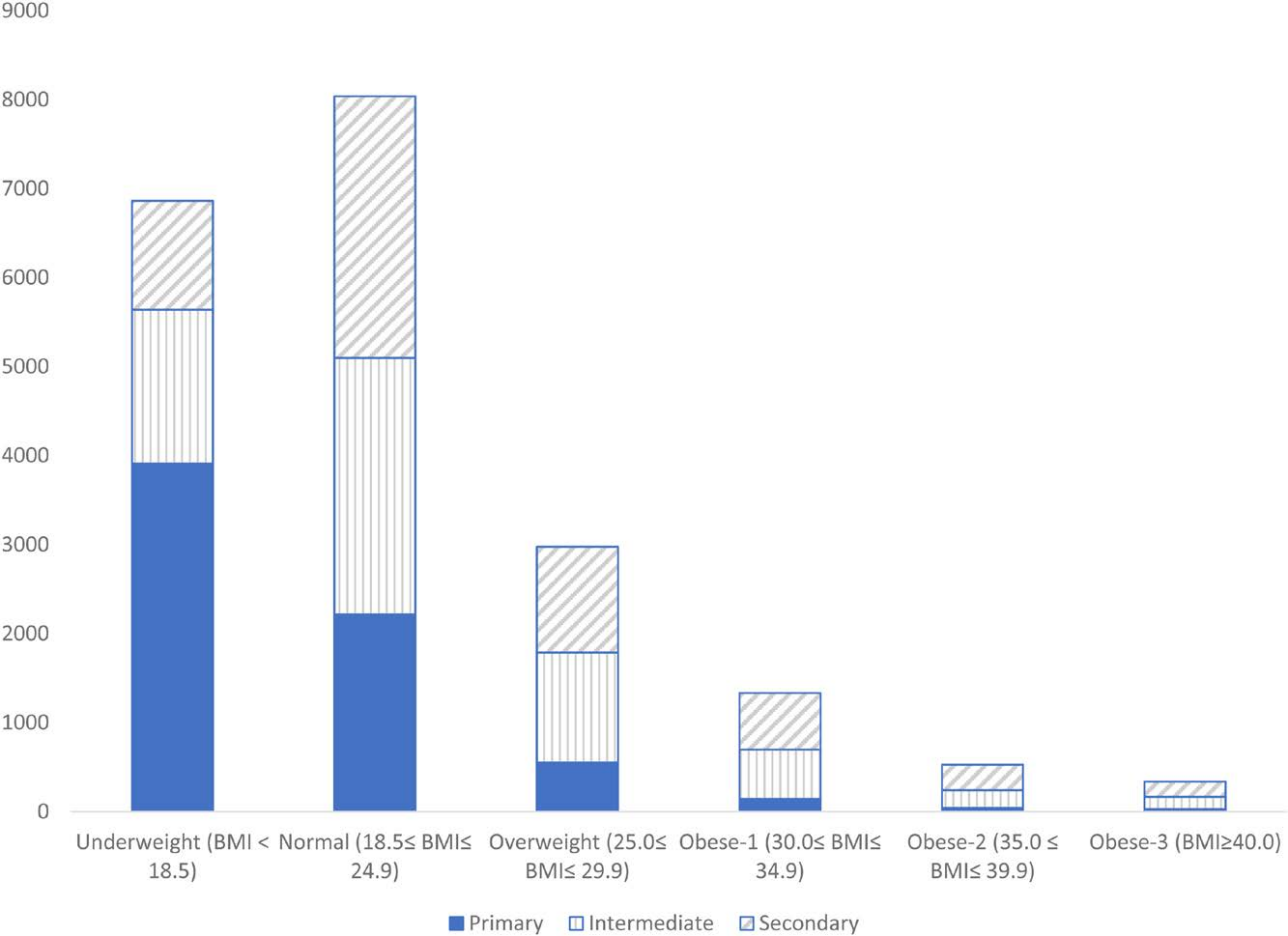
Comparison of the distribution of BMI classes across education levels, school health campaign. BMI = body mass index.

Table 5 and the accompanying Figure 2 show the comparison of the distribution of BMI classes across education levels. The table shows that there is a significant association between BMI and school level (*P* < .001). For all the school levels, the proportion of obese or overweight is much lower than the proportion of normal or underweight. Also, for the overweight and obese categories, the proportions decrease for all levels of education.

**Table 5:** Association between school level and education level, school health campaign.

BMI classes	Primary	Intermediate	Secondary	χ^2^(df)	*P* value
Underweight (BMI < 18.5)	3910	1729	1223	2737.76 (10)	<.0001
Normal (18.5 ≤ BMI ≤ 24.9)	2216	2882	2941		
Overweight (25.0 ≤ BMI ≤ 29.9)	554	1234	1186		
Obese-1 (30.0 ≤ BMI ≤ 34.9)	144	553	637		
Obese-2 (35.0 ≤ BMI ≤ 39.9)	43	198	288		
Obese-3 (BMI ≥ 40.0)	28	138	169		
Total	6895	6734	6444		

BMI = body mass index, df = degree of freedom.

The result of the multivariate regression analyses is shown in Table 6. The overall model fit was significant; F(5, 20,067) = 654, *P* < .001. However, the predictive power of the model is relatively low as the adjusted *R*^2^ is about 14%. After controlling for the other variables in the model, sex drops in significance. The remaining significant variables were education level, age, glucose level, and blood pressure.

**Table 6:** Multiple regression with BMI as outcome variable, school health campaign.

Dependent variable	BMI_2				
N	20,073				
Multiple *R*	0.37433				
Multiple *R*^2^	0.14012				
Multiple *R*^2^adj	0.13991				
ANOVA					
F	654.01				
df1, df2	5, 20,067				
*P*	0				
	Coefficients	Standard error	t	*P* value	*R* ^2^
Constant	11.794	0.32296	36.519	<.001	
Sex	0.144	0.085	1.694	.090	0.001
School level	2.006	0.072	28.029	.000	0.103
Age	0.199	0.023	8.721	.000	0.069
Glucose level	0.012	0.002	7.809	.000	0.004
Blood pressure	1.159	0.044	26.584	.000	0.033

BMI = body mass index, df = degree of freedom, *R* = regression, *R*^2^adj = adjusted *R*^2^.

## 4. Discussion

This large, representative school-based study found that the percentage of overweight or obese among high school children in the Eastern Province of Saudi Arabia was 25.7%. This finding is consistent with another study conducted in other regions; Saudi Arabia showed that the prevalence of obesity was higher in high schools.^[16,17]^ To date, several studies on childhood obesity among school students were conducted in the Kingdom of Saudi Arabia; however, these studies were small, cross-sectional studies. Al-Shammari et al (2001) performed a cross-sectional study on a population-based sample of Riyadh city in the Central Province that was not randomly chosen. In total, there were 4775 participants, of whom 1848 were children and 2927 were adults. Among the children, about 10.5% were overweight, and 8.7% were considered obese.^[18]^ In the same region, a new study reported a higher prevalence rate of obesity (15.7%) among school-age students.^[19]^ A study in Jazan (Southern Province) reported that the prevalence of obesity and overweight was 12.4% and 10.1%, respectively.^[20]^ In contrast, according to a study carried out by Qamar Farshori et al^[21]^ in 2015, it was found that ≈48% of male children were either overweight or obese, and for female children, 13% of them were obese and 20% were overweight.

The present study found no significant gender differences regarding BMI classes. However, some local studies reported inconsistent results regarding the obesity and gender difference prevalence among the Saudi population. Results from other local studies in this regard, however, were conflicting.^[17,22]^ According to a previous review, results suggested that the gender differences in obesity rates are generally small and inconsistent.^[10,23]^

Our study also investigated some obesity-related risk factors and found that the risk factors for obesity in this study population were age, education level, age, glucose level, and blood pressure. The high prevalence of elevated blood pressure or hypertension among the study’s students is in agreement with a similar study conducted in the same region of Saudi Arabia.^[24]^ The relation between elevated blood pressure and obesity is well documented. Previous studies showed that there is approximately 3-fold increased risk in obese children for developing hypertension rather than nonobese children.^[25,26]^ Indeed, previous research from the Eastern Province of Saudi Arabia demonstrated that behavioral aspects could explain the probable cause of the increase in BMI and blood pressure in early life. These factors included physical inactivity, eating habits, especially high salt intake,^[27]^ fructose aerated beverages and energy drink consumption,^[28]^ physical inactivity,^[29]^ and obesity.^[15]^ In addition, increased TV viewing, use of smart tools, and sleep duration at a young age have been linked to an increased risk of being obese.^[30,31]^

There is evidence that the prevalence of obesity in school age is increasing dramatically in the Kingdom of Saudi Arabia, possibly as a result of a sedentary lifestyle and urbanization, and the problem appears to be becoming worse as the children become older.^[31]^ Urbanization is considered a major risk factor for obesity in developing countries in economic transition.^[32]^ The cities of Saudi Arabia are now modernized, with separate zoning and large transportation networks for commercial areas and residential areas. This type of city design requires the use of the automobile for most trips and totally discourages walking, which reflects negatively on public health.^[33]^ Moreover, the access to technology (e.g., smart phone, video games, and computers) is likely to contribute to a more sedentary lifestyle with significant association with obesity among Saudi children.^[34]^

## 5. Conclusion

Our evidence clearly shows that childhood obesity is still a growing problem in Saudi Arabia and that children are at risk of becoming overweight and obese with age. In conclusion, levels of overweight children and obesity among high school children were found to be 25.7% in the Eastern Province of Saudi Arabia. The high prevalence of overweight and obesity, as well as high levels of BP, suggested implementing appropriate lifestyle interventions program to readjust this problem among childhood. This requires the development of a school health information system in which there is routine collection of health-related variables, such as BMI, blood pressure, and glucose level. Future studies should include associated factors such as social background, exercise, culture, nutrition, and genes.

### 5.1. Strength and limitation

This is a multicenter school-based screen that used a representative and large sample of over 20,000 students. The study had a very high participation rate. Limitation of the study includes the fact that blood pressure readings were taken from a single reading in 1 visit.

## Acknowledgments

The authors gratefully acknowledge the support of the present from the Saudi Diabetes and Endocrine Association. Partial support from the General Directorate of Education Eastern Province is also gratefully acknowledged.

## Author contributions

Conceptualization: Waleed Albaker.

Formal analysis: Sulaiman Bah.

Investigation: Rim Saklawi, Kamaluddin Motawei.

Project administration: Basem Futa.

Supervision: Waleed Albaker.

Validation: Sulaiman Bah.

Writing—original draft: Waleed Albaker.

Writing—review and editing: Mohammed Al-Hariri.
